# Metachronous Bilateral Silent Sinus Syndrome: A Case Report

**DOI:** 10.22038/ijorl.2020.42809.2396

**Published:** 2020-05

**Authors:** Paolo Farneti, Andrea Bellusci, Alfredo Parmeggiani, Ernesto Pasquini

**Affiliations:** 1 *Bologna University Medical School - Sant’Orsola-Malpighi Hospital, Via G. Massarenti 9 40138 Bologna, Italy.*; 2 *Azienda Unità Sanitaria Locale di Bologna, ENT Department Bologna, Via Altura 3 40139 Bologna, Italy.*

**Keywords:** Diplopia, Enophthalmos, Endoscopy, Maxillary Sinus; Orbit, Paranasal Sinuses

## Abstract

**Introduction:**

Bilateral silent sinus syndrome (SSS) is a very rare pathology reported only in few papers in literature. Most of the described cases are simultaneous, and only one had a metachronous presentation. The evolutionary phases of the disease have yet to be well demonstrated and a complete radiological evaluation is needed to demonstrate the pathogenetic mechanisms that cause the disease.

**Case Report:**

A 45-year-old male presented with a left SSS and a bilateral concha bullosa. He developed a contralateral SSS two years after an endoscopic uncinectomy and re-ventilation of the diseased maxillary sinus. This case is the second reported in literature with a metachronous presentation. A pure endoscopic approach has led to the resolution of symptomatology and the full restoration of the ventilation of the maxillary sinuses. The key role of the uncinate process in the genesis of the pathology has been well demonstrated by the onset of a contralateral SSS in a normally developed maxillary sinus thanks to a complete radiological follow-up.

**Conclusion:**

Bilateral presentation is a rare entity; however, it should be considered in patients with SSS. A minimal endoscopic uncinectomy could also prevent the onset of the disease on the healthy side.

## Introduction

The definition of silent sinus syndrome (SSS) was coined by Soparkar in 1994 to define a rare disease characterized by spontaneous enophthalmos and hypoglobus secondary to ipsilateral maxillary sinus hypoplasia and orbital floor collapse unassociated with other nasal symptoms (1). After this classification, bibliographic reports have progressively become more abundant in journals of various specialties, namely otorhinolaryngology, ophthalmology, maxillofacial, radiology. In recent years, reports have increased further, and this has led to a more precise identification of the unresolved aspects of this disease. Bibliographic reports of bilateral SSS are very rare. A review of the literature has allowed to identify only a few papers on this topic (2-7).

We present a case of metachronous bilateral SSS with the appearance of contralateral localization two years after surgery in the first maxillary sinus. The evolution in radiological findings during the follow up has allowed to formulate pathogenic hypotheses on this rare disease and confirm how the restoration of good sinus ventilation could lead to re-expansion of the sinus cavity, restoration of the orbital floor, and a repositioning of the eyeball without a need for a simultaneous repair of the orbital floor. We have therefore compared our case with those described in previous studies in order to better define the possible pathogenic mechanisms, the different evolutionary phases, and the best surgical treatment.

## Case Report

A 45-year-old male was referred to our center for changes in his facial appearance with ocular asymmetry started 3 months before. No previous records of trauma or surgical procedures were reported in paranasal sinuses. The patient never complained of symptoms of acute or chronic rhinosinusitis. The clinical and ophthalmological examination revealed a 3 mm left enophthalmous and hypoglobus with neither visual deficits nor diplopia.

The patient underwent a computed tomography (CT) of paranasal sinuses which showed opacification and hypoplasia of left maxillary sinus, lateralization of the uncinate process, and depression of homolateral orbital floor. A bilateral concha bullosa of the middle turbinate was also present (Fig’s1,2:1A-2A). Moreover, left SSS was diagnosed, and the patient underwent functional endoscopic sinus surgery. Antrostomy was performed with retrograde uncinectomy together with the aspiration of mucoid secretion in the left maxillary sinus followed by the removal of the lateral lamella of the concha bullosa of the middle turbinate on both sides. The patient showed the regression of the aesthetic defect and improvement of the left enophthalmos and hypoglobus six months after surgery. A post-operation CT scan was performed 10 months after surgery and allowed to find a complete re-ventilation of the left maxillary sinus with normalization of the maxillary sinus walls which appeared thin and demineralized before the intervention. On the right side instead, an initial and mild lateralization of the uncinate process could be identified with a still well-ventilated maxillary sinus (^Fig’s 1^,2: 1B-2B).

After 24 months post-surgery, the patient underwent a CT scan for other reasons, and a complete right maxillary sinus opacification was observed with lateral displacement of the uncinate process and a thinning of the maxillary sinus bone walls, especially the posterior and superior ones. A lowering of the orbital floor was also present, compared to the contralateral side; therefore, diagnosis of right SSS was made (Fig’s 1,2:1C-2C).

Right endoscopic uncinectomy and middle meatotomy were performed, and marked implosion of the sinus walls was found with the presence of dense mucoid secretions. A CT scan performed after 5 years showed well-ventilated maxillary sinuses, bone reposition in the orbital floors and maxillary sinus walls, partial re-expansion of the sinuses, and repositioning of the eyeballs at the same level (Fig’s 1,2;1D-2D).

**Fig 1:**
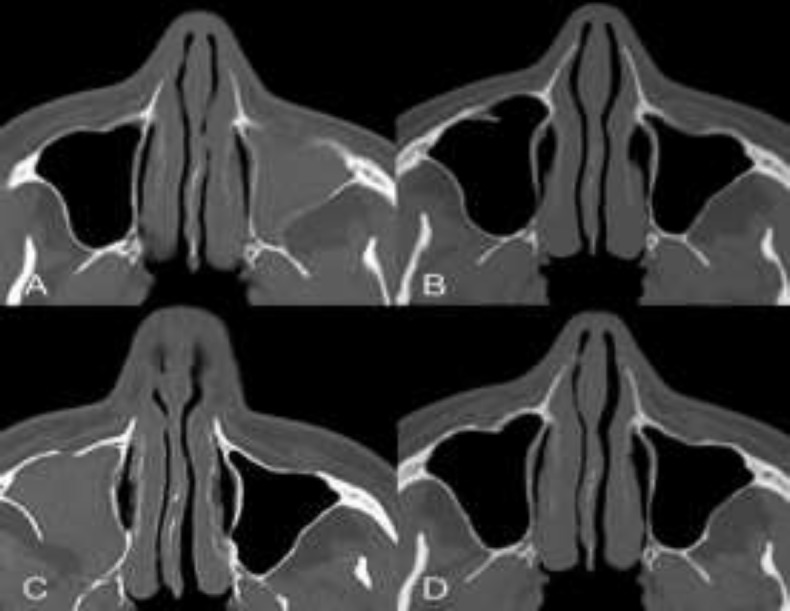
A)**:** Axial computed tomography scan showing pre-operatory left silent sinus syndrome with left retro-antral fat pad expansion and demineralization of the anterior and lateral walls of the maxillary sinus, B) Left maxillary sinus re-ventilated ten months after the first surgical operation, C) Occurrence of right silent sinus syndrome with retroantral fat pad expansion and thinning of the posterior wall after 2 years, D) Post operatory scan 5 years after the second surgical operation

**Fig 2:**
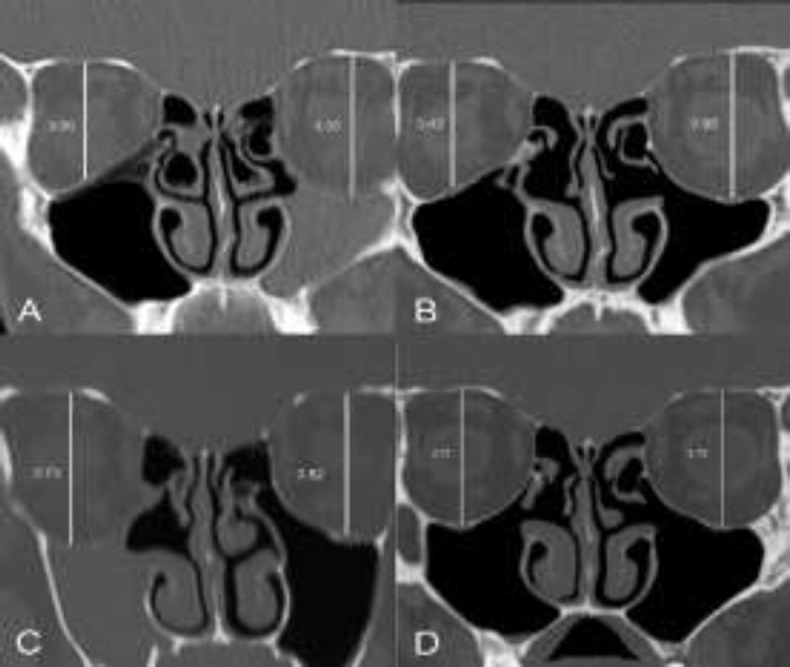
Coronal computed tomography scans showing the vertical dimension in centimeters of each orbit to demonstrate the grade of lowering of the maxillary sinus roof, A) Left silent sinus syndrome with uncinate process lateralization and maxillary sinus opacification before surgical operation. Evidence of a bilateral concha bullosa, B) Left maxillary sinus re-ventilated and bilateral concha bullosa correction. It is possible to notice a slight lateralization of the right uncinate process ten months after the first surgical operation, C) Evidence of right silent sinus syndrome with sinus opacification and lowering of the orbit two years after the first surgical operation, D) Post-operative computed tomography scan showing the re-ventilation of both maxillary sinuses 5 years after the second surgical operation. The orbital floors are at the same level on both sides

## Discussion

Kass et al. (1997) in an extensive series of 22 cases of chronic maxillary atelectasis presented 2 bilateral cases that could not be considered true SSS for the association with sinonasal symptoms, the absence of bone wall deformity, and hypoglobus/enophthalmos (8).

In 2011, Liss reported a case defined as bilateral SSS (2); however, according to the classical definition (1), even this case could not be considered SSS since the patient had previously undergone surgery and chemoradiation for an undifferentiated nasosinusal carcinoma. The pathology would be better defined as chronic maxillary atelectasis.

The first case of bilateral SSS with simultaneous involvement of two maxillary sinuses is the one described by Suh et al. in 2012 (4). Complete bilateral opacification of the maxillary sinuses was found during a cranial CT scan performed for other reasons with collapse of the sinus walls and in particular of the orbital floor, responsible for enophthalmos. This allowed the diagnosis of bilateral SSS. The authors pointed out that in cases of contemporary bilateral onset, it is more difficult to clinically diagnose the pathology since the presence of bilateral enophthalmos and hypoglobus does not lead to obvious facial asymmetries. In these cases, therefore, diagnostic confirmation can only come from radiological findings.

In the same vein, Ferri et al. (2012) reported a case of left SSS with a history of left eye enophthalmos with double vision and severe deepening of the superior palpebral sulcus. According to the results, the case was surgically treated with simultaneous endoscopic left uncinectomy, maxillary antrostomy, and reconstruction of the orbital floor with Medpor through a subciliary incision (3). The patient presented a similar symptomatology four months after surgical treatment of left SSS on the right side with enophthalmos and hypoglobus associated with a lateralization of the right uncinate process and right middle turbinate, an opacified right frontal sinus, inflammation of the maxillary sinus, and a thinned and downward-retracted floor of the orbit on the same side. The patient underwent surgery with the same previous technique. Clinical resolution of symptoms was achieved 3 weeks after surgery and confirmed with the CT scan obtained four months later (3).

The authors underlined how it is possible to appreciate the evolution from a normal maxillary sinus to the radiological signs typical of SSS in just four months. They also claimed that the possibility of a delayed bilateral presentation imposes a proper counselling of the patient and a strictly close follow-up.

In a study conducted by Su. et al. in 2013, a case of bilateral SSS was treated for the first time with balloon dilatation of maxillary ostium as an in-office procedure (5).

Similarly, Gunaratne et al. (2016) described a case of bilateral chronic maxillary atelectasis which was considered as an early stage of bilateral SSS due to the absence of evident clinical signs. The patient was treated with a bilateral uncinectomy and maxillary antrostomy (6). One last case of a concomitant bilateral SSS was presented in 2018 by Trope et al. and treated by maxillary antrostomy and total ethmoidectomy without orbital floor reconstruction (7).

The case presented in this case report is very similar to that in the study performed and published by Ferri et al. in terms of clinical presentation (3). The second location of our case appeared about 2 years after the first one and with the same radiological manifestations of the first site. The presence of a concha bullosa could have led to the lateralization of the uncinate process on the left side while a minimal trauma to the uncinate process on the right side during the removal of the concha bullosa could have led to the destabilization of an already altered uncinate process.

The role that the uncinate process can play in the pathogenetic mechanism of the SSS has been hypothesized by Kass in 1998. He claimed that a deficient posterior attachment of the uncinate process could lead to a flap valve occlusion of the natural ostium, gas resorption, negative pressure in the sinus, and accumulation of fluids (9). This same pathogenetic theory has been resumed and confirmed by Hazan et al. and Illner et al. (10,11). In 2008, Bussolesi and Castelnuovo suggested that a large floppy uncinate process closely adjacent to the lateral nasal wall might provoke a one-way air out-flow pneumatic valve mechanism within the osteomeatal complex leading to the atelectasis of the maxillary sinus. This phenomenon could be elicited by some physiological respiratory actions which produce negative pressure in the sinuses (12).

It is interesting to note that in the case we have described, the initial lateralization of the right uncinate process documented since the first postoperative CT scan could demonstrate how this was the initial pathogenetic mechanism of the SSS that would have occurred the following year. This confirms the theory of other authors (3,11,12) and demonstrates the possible rapid onset of the maxillary sinus collapse.

We performed only endoscopic middle meatotomy with the result of complete resolution of the clinical symptomatology. Postoperative CT examination documented the re-ventilation and re-expansion of the maxillary sinuses and replacement of orbital floor. Functional endoscopic sinus surgery represents the gold standard for re-ventilation of the diseased antrum and restoring sinus function in SSS (12-14). 

One remaining open question would be the following: Would it be advisable to perform also a contralateral uncinectomy in patient with unilateral SSS? The answer will be probably “not always”; however, we should be aware of the possibility that the same pathology can also develop on the other side. An adequately informed consent to the patient should also be advisable in order to take the best therapeutic decision.

## Conclusion

Regarding the recent bibliographical orientations, the case just presented, and those we have previously published (13,14), it has been proven that a pure endoscopic uncinectomy and middle meatotomy is effective in guaranteeing the resolution of both the enophthalmos and the aesthetic defect present in SSS. The risk of developing SSS also on the contralateral side should sensitize the surgeon to evaluate whether or not to perform a minimal uncinectomy on the healthy side. 
